# Promoting healthy lifestyle behaviours in the preschool setting: perceptions and needs of teachers and principals

**DOI:** 10.1186/s12889-025-24379-4

**Published:** 2025-09-09

**Authors:** Ellinor Nilsson, Hanna Tigerstrand, Christine Delisle Nyström, Emmie Söderström, Christina Alexandrou, Marie Löf

**Affiliations:** 1 Department of Medicine, Huddinge, Karolinska Institutet, 141 83 Huddinge, Sweden; 2 Department of Health, Medicine and Caring Sciences, Linköping University, 581 83 Linköping, Sweden

**Keywords:** Early childhood education, Eating behaviour, Health promotion, mHealth, Movement behaviours, Physical activity, Preschool children, Qualitative, Screen time, Sleep

## Abstract

**Background:**

Preschools are important environments in shaping young children’s lifestyle behaviours, including movement (physical activity, screen time, and sleep) and eating behaviours. Few studies have investigated how teachers and principals can be supported in promoting healthy lifestyle behaviours in the preschool setting and whether a digital support tool could be a way forward. This study aimed to explore preschool teachers’ and principals’ perceptions, needs, and prerequisites for promoting healthy lifestyle behaviours, as well as their preferences for a potential digital support.

**Methods:**

In 2024, ten teachers and five principals from five regions across Sweden were recruited through purposive sampling and semi-structured interviews were conducted through video calls. Analysis was guided by the research aims and conducted using inductive content analysis.

**Results:**

Teachers as well as the preschool environment were highlighted as crucial for promoting physical activity in children. Teacher involvement was found to be central for motivating children to be physically active, especially children who do not spontaneously engage in physical activity. Communication between preschools and parents was also considered important for promoting healthy lifestyle behaviours. Furthermore, participants emphasized the value of face-to-face support such as lectures or workshops, potentially complemented by a digital tool, for increasing motivation and awareness among educators. Such combined support was highlighted as particularly useful for educators with low interest in physical activity. Finally, the sections on physical activity in the preschool curriculum were perceived as unclear and participants expressed a need for clearer guidance.

**Conclusions:**

Findings suggest that face-to-face support in combination with a digital tool can facilitate the promotion of physical activity and other healthy lifestyle behaviours in the preschool setting. Also, clear guidance within the curriculum and policies are warranted.

**Supplementary Information:**

The online version contains supplementary material available at 10.1186/s12889-025-24379-4.

## Background

Healthy movement (i.e., physical activity, screen time, and sleep) and eating behaviours are important for children’s health and development [1]. The World Health Organization (WHO) recommends that preschool-aged children: (i) accumulate at least 180 min of physical activity daily including 60 min being moderate-to-vigorous physical activity; (ii) limit sedentary screen time to no more than 60 min per day; and (iii) obtain 10–13 h of good-quality sleep [2]. A recent pooled analysis of global data including 7017 three- to four-year-old children across 33 countries found that 49%, 42%, and 81% met the guidelines for physical activity, screen time, and sleep, respectively [3]. Furthermore, Chong et al. observed that only 14% of pre-schoolers met al.l three guidelines [3]. Similarly, recently published representative data from Swedish children aged 4–17 years found that only 37% of children aged 4–6 years met the daily recommendation for physical activity [4]. Moreover, 14% of parents reported that their 4-6-year-old had at least three hours of screen time the previous day [4]. In terms of eating behaviours in this age group, recent dietary data from a representative sample of Swedish four-year-olds found that less than one in ten met the recommendation of at least 400 g of fruit and vegetables per day [5]. These findings highlight significant gaps in meeting health guidelines among young children. Given that almost 87% of Swedish children aged 1–5 years spend a considerable part of their day in preschool [6], this setting is an important arena for children’s development and health behaviours and provides a valuable opportunity for health promotion early in life [6–9].

The WHO Global Action Plan on Physical Activity 2018–2030 [10] calls for childcare providers to actively promote physical activity within early childcare settings. Given their role as key stakeholders in the preschool setting, teachers and principals have the potential to promote healthy movement behaviours in young children, making them a crucial group to target. However, evidence-based and concrete tools to support preschool educators in promoting healthy movement behaviours in young children are lacking.

Previous interventions to support preschool teachers in promoting healthy lifestyle behaviours in children through face-to-face training have shown no or small effects [11, 12]. For instance, Leis et al. [11] conducted a randomized controlled trial aimed to increase physical activity levels and improve healthy eating behaviours in preschoolers through on-site training for childcare educators. The intervention marginally increased children’s fruit and vegetable intake and no effect on physical activity levels were observed [11]. Additionally, Mavilidi et al. [12] conducted an intervention where early childhood educators in Greece were trained through four face-to-face sessions over four weeks to promote physical activity in three- to five-year-old children. Physical activity was assessed with pedometers; however, no significant differences between experimental groups were found. It is possible that few face-to-face education sessions for pre-school teachers is not enough to increase children’s physical activity levels and that more continuous support is warranted.

Digital tools can provide cost-effective, scalable solutions that are easily accessible across regions, and have shown promise in supporting healthy movement behaviours in preschool-aged children [13, 14]. Nevertheless, existing digital interventions have mainly targeted parents rather than preschool teachers and to our knowledge, there are only two digital interventions targeting preschool teachers [15, 16]. Hoffman et al. [15] conducted a pilot cluster randomized controlled trial, where teachers received a four-week online course to improve their knowledge, skills, and confidence in promoting moderate-to-vigorous physical activity in preschool children. Children whose teachers had taken the course significantly increased their moderate-to-vigorous physical activity during school hours, while children whose teachers were in the control group did not [15]. Another teacher-targeted pilot intervention was conducted by Byun et al. [16], where preschool teachers digitally monitored children’s accelerometer-derived physical activity levels and provided more possibilities for physical activity in the classroom if needed. Furthermore, they found that children in intervention preschools had significantly less sedentary time and higher physical activity levels than children in control preschools [16]. These findings suggest that digital solutions have the potential to be an effective tool for preschool teachers in promoting healthy lifestyle behaviours; however, there is a necessity to better understand preschool teachers’ needs in order to develop an optimal tool for them. Therefore, the aims of this study were to explore preschool teachers’ and principals’ perceptions, needs, and prerequisites for promoting healthy lifestyle behaviours, as well as their preferences for a potential digital support to promote these behaviours in preschool aged children. The concept of a digital tool was introduced as a hypothetical idea to explore the participants’ views on whether such a tool would be considered useful and desirable.

## Methods

### Setting

In Sweden, preschools are publicly funded and available for children from age one until six years of age when they start compulsory school. Approximately 87% of children 1–5 years old attend preschool, making it a central part of early childhood education in Sweden [6]. Preschools are governed by the Education Act (2010:800), of which the national preschool curriculum is a part of [17]. The preschool curriculum highlights the importance of play, equality, democratic values, and the development of children’s social, emotional, and cognitive skills [17]. It includes goals related to health and well-being, as well as guidelines that all preschools should follow. For example, the guidelines include that preschool teachers are responsible for ensuring that all children experience that learning is fun, are provided opportunities that stimulate new knowledge and experiences, and are supported in their play, development and learning [17]. The guidelines also say that all children should use digital tools in a way that stimulates development and learning and should be offered a balance between active and calm activities [17].

In the Swedish preschool system, each preschool is led by a principal who is responsible for the management, pedagogy, staff, and ensuring that the preschool follows the Education Act and curriculum. One principal can be in charge of several preschools. Each municipality implements the national policies in their preschools, which may lead to some differences in the daily operations and resources across municipalities.

In all Swedish preschools, meals are provided throughout the day without any additional cost. This always includes snacks and a cooked lunch, and caregivers can often pre-order breakfast for their child, depending on what time their child starts. Children typically spend a large proportion of their weekday in preschool [6]. Often, they are dropped off at preschool in the morning and picked up in the late afternoon, but some children may be picked up already around lunch time. Nevertheless, most children spend many hours in preschool each week, making it a key environment for shaping children’s lifestyle behaviours.

## Participants

Prior to initiating the current study, a stakeholder analysis was performed to identify key players and understand their interests and influence in the potential development of a digital tool for preschool teachers. The direct stakeholders that were identified were preschool teachers and children, while the indirect stakeholders were preschool principals, parents, researchers, and policymakers at the municipal, regional, and national levels, working with children’s health. Among the identified stakeholders, preschool teachers and principals were found to have the greatest insight into preschool work and the potential for implementing changes. Therefore, they were the target group for this qualitative study.

Participants were recruited through preschools participating in the Swedish arm of the International Study of Movement Behaviours in the Early Years (SUNRISE) study [18]. Purposive sampling was utilized to recruit preschool teachers and principals from different regions and municipalities across Sweden, representing both urban and rural areas as well as high and low socioeconomic areas, based on the location of the preschool [19]. Emails were sent by the research team to teachers and principals asking if they would be interested in participating in an interview study on children’s lifestyle behaviours in the preschool environment. If they were interested in participating, they were provided with full study information and informed consent via email and post. Upon receiving their signed informed consent, a member of the research team contacted them to schedule an interview via video call. A total of 18 teachers and principals were contacted and invited to participate in the study on a rolling basis. Three declined to participate after receiving full study information; one declined due to illness, one did not provide a reason, and one did not respond. Recruitment continued until data saturation was reached, i.e. when broad and rich data to be able to answer the study aims had been obtained. A total of 15 teachers and principals from five different regions in Sweden (Dalarna, Skåne, Stockholm, Södermanland, and Östergötland) participated.

Based on education level, nine participants worked in preschools located in high socioeconomic status areas and six in low socioeconomic status areas [19]. These included ten preschools in urban areas and five in rural areas. To be included in this study, the teachers and principals needed to be currently working at a preschool in Sweden and there were no exclusion criteria. The interviews were conducted between April and June 2024. The consolidated criteria for reporting qualitative research (COREQ) [20] were used in the reporting of the current study. The Swedish Ethical Review Authority approved this study (reference number 2023-06666-01), and all participants provided written and verbal consent before the interviews were conducted.

## Data collection

All interviews were performed over a video call by HT, a researcher with a background in physiotherapy. As the principals and teachers in this study were recruited from preschools in the Swedish arm of the SUNRISE study [18], there was a prior relationship; however, only in relation to planning data collection at their respective preschool. At the beginning of each interview the respondent received information on the interview process as well as an explanation of the aims of the study. Thereafter, the respondent was asked for verbal consent and permission to audio record the interview. The interview guide (Additional file 1) was based on 15 core questions developed by EN (PhD student and nutritionist) and discussed and revised by all authors. The same core questions were asked to all participants, and probing questions were asked based on the individual responses. As part of the process, field notes were taken after each interview to facilitate reflection, but they were not used in the formal analysis. The audio was transcribed verbatim by a professional transcribing service. The interviews ranged between 26 and 49 min (average 36 min). Recruitment and data collection continued until perceived data saturation was reached and a rich material was obtained to answer the aims of the study.

### Data analysis

The data was analysed using content analysis with an inductive latent approach [21, 22]. At first, EN and HT listened to and read transcripts of all the interviews, followed by an independently performed initial manifest coding [21, 22]. This was done through an iterative and data-driven procedure, keeping the research aims in mind and with a low degree of interpretation. Thereafter, EN conducted the latent coding to uncover underlying meanings [21, 22]. EN and HT further condensed the codes into preliminary categories and reflected upon these until reaching agreement. Subsequently, EN organized and combined the categories into groups that revealed potential themes and sub-themes and reviewed these with HT. Feedback on the potential themes and sub-themes was provided by ES and CA, researchers and nutritionists with experience in qualitative analysis. Finally, EN together with HT had a concluding discussion about the revised themes until reaching consensus on final labelling. While sub-themes were identified during the analysis, we decided to not present them in the results to maintain focus on the main themes.

## Results

**Fig. 1:**
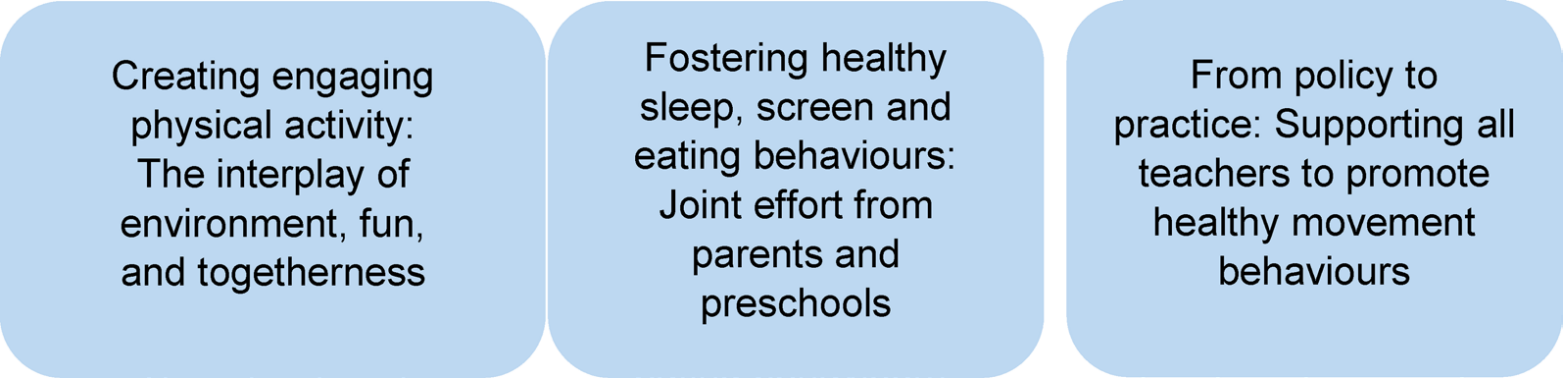
Overview of the themes generated from the analysis of interviews with preschool teachers and principals

Among the 15 participants, 13 were women, two were men and the age ranged from 31 to 64 years (mean 49 years). Three teachers worked at preschools with an outdoor profile, meaning that they spend almost the whole day outdoors. There were ten teachers and five principals; however, three teachers had previously worked as a principal. The work experience of participants was 5–38 years (mean 19 years). Two had completed upper secondary education and 13 had completed higher education. Three themes were identified and are presented in Fig. 1. Quotes from the interviews, labelled with the respondent’s profession are included as a support to the themes.

### Creating engaging physical activity: the interplay of environment, fun, and togetherness

Physical activity in children aged 3–5 years was described as a combination of activities of low and high intensity, where playing was highlighted as the children’s main source of physical activity. Spontaneous movement and planned activities were emphasized as important, and having physical activity as a part of everyday life was highlighted as essential. Outdoor play, active transport and activities led by teachers were reported as important contributors to children’s physical activity. Furthermore, the participants reported that breaks from sedentary time were necessary for the children’s well-being. Encouraging every child, including those that do not enjoy physical activity, was considered fundamental and teachers emphasized that being supportive is crucial for helping these children form healthy physical activity behaviours that can last into adulthood.

I take a lot from my own experience, I wasn’t encouraged as a child to move and go to sports or activities, and I have a really hard time getting started today… and I want people to understand that.—Teacher 6.

Indoor areas were emphasized as being dedicated to calm, often sedentary activities, and a common negative view on engaging in physical activity of higher intensities indoors was reported. Moreover, physical activity in the indoor environment was described to be affected by limited space. This was partly due to reconstruction of larger playing areas into smaller rooms, limiting the possibilities to engage in moderate-to-vigorous physical activity. In contrast, some stated that the indoor environment does provide opportunities for physical activity, but to a limited extent.

There were discrepancies regarding how moderate-to-vigorous physical activity is achieved. Some viewed spontaneous play outdoors in open spaces, e.g., parks, as a prerequisite for moderate-to-vigorous physical activity, while others emphasized the importance of organized exercise, initiated and supported by teachers. Their support for moderate-to-vigorous physical activity was considered especially important for children who do not naturally enjoy physical activity. Also, the importance of teaching children that elevated heart rate and feeling tired are not harmful was emphasized. Without this understanding and encouragement from teachers, it was reported that children commonly perceive these sensations as dangerous, causing them to interrupt their physical activity before reaching higher intensities. The findings also revealed that when teachers actively participate in physical activity, they motivate children to be active for longer periods of time, increasing the likelihood of reaching higher intensities. Furthermore, it was expressed that children find physical activity together with teachers more enjoyable, which also was considered a vital part of getting children active.

If you have a person [teacher] who enjoys being physically active in their free time, maybe has an interest in it, then they think it is important, and they will probably feel ‘we need to do this and that’ […] I think it is very much up to each adult on an individual level.—Teacher 3.

Teachers and principals were aware that children require a lot of physical activity but expressed difficulties in knowing exactly when children have met their individual needs. Some stated that the majority of a child’s day should consist of physical activity and that both spontaneous and organized physical activity are important for their needs. It was highlighted that being outdoors most part of the day is necessary to get enough physical activity, and that preschool yards are often too small to support this. Consequently, it was believed that children must leave the preschool premises to engage in adequate physical activity, and that this is not done frequently enough.

Another raised barrier for physical activity was lack of resources in terms of staff and budget, making sedentary activities more convenient.

Often on days when we are understaffed and can’t get substitutes, they [teachers] cancel outdoor activities because it takes too much time to dress 24 children and take them outside. It becomes too much of a hassle.—Principal 1.

Understaffing also limited the possibilities for taking children on field trips, and with poor economic resources, the possibilities of improving the indoor and outdoor environment were reported as limited. It was also requested to invest in equipment that children can use to be physically active without help from adults, which could support preschool teachers in promoting more physical activity when understaffed.

## Fostering healthy sleep, screen and eating behaviours: joint effort from parents and preschools

Preschools were highlighted as important environments for fostering healthy lifestyle behaviours in young children, influencing not only physical activity but also screen time, sleep, and eating behaviours. However, since these behaviours extend beyond the preschool setting, teachers and principals emphasized the importance of maintaining a dialogue with parents to ensure consistency between the home and preschool environments.

Participants emphasized a recent shift in attitudes towards screen use in preschools due to parental concerns and societal debates. While they had previously been pressured to integrate screens into preschool routines, they were now encouraged to limit screen activities. It was also observed that children often prefer traditional media and adult interaction over screens. As an example, teachers raised that reading books aloud is more appreciated than listening to audiobooks with accompanying images on a large screen. Nevertheless, the use of screens in preschools was described as mostly dependent on teachers’ perspectives and attitudes. Participants also stated that when using screens, it should have a clear learning purpose and be kept brief, with some advocating for minimal screen use in preschool activities.

We have guidelines that there must be a purpose for all digital tools. For example, if we are going to use a movie, it must have a purpose related to the project we are currently working on.—Principal 5.

While participants acknowledged that screens can be useful, they stressed that screens should not replace analogue learning experiences, and that it is not a responsibility of preschools to teach children how to use screens. Furthermore, children should be encouraged to be producers rather than passive consumers, and creative apps were thought to be valuable, provided that a teacher was present. Screens were also seen as potential promotors of physical activity when used appropriately.

Teachers and principals believed that screen time is most problematic in the home environment, where children may remain seated in front of a screen for extended periods, particularly on weekends. Some participants believed that parents might feel more comfortable allowing screen time at home if their child has been active at preschool.

Furthermore, adequate sleep was considered crucial for children’s emotional regulation, especially for younger preschoolers. Teachers reported that long preschool days require sufficient recovery, but that all children do not get enough sleep at home, resulting in challenging days for children as well as teachers. Moreover, teachers and principals reported that some parents do not permit daytime sleep in order to encourage earlier bedtimes at home, while others rely on preschool naps to ensure that their child gets enough sleep. Respondents emphasized the importance of educating parents about the necessity of sleep to ensure that all children get sufficient sleep. For children who do not nap, downtime, i.e., rest, was still considered important and was included in the daily schedule, often as listening to stories. Some expressed that it is important that children not only rest but also learn the skill of how to do it and why it is important.

They learn some form of winding down and relaxation to be able to recover in a good way, without just being in front of a screen or needing to be occupied with something else.—Principal 2.

In terms of healthy eating behaviours, all preschools serve lunch and snacks and some also serve breakfast. These meals were expressed as essential, especially since eating behaviours in the home environment may vary, making the role of teachers in fostering healthy eating behaviours crucial. Preschool meals were also considered particularly valuable for children whose parents lack time for preparing healthy meals at home.

We work with them. We sit with them. We eat the same food they eat. And we sit together with the children, and it is calm and a pleasant time. We also believe this is very important.—Teacher 2.

Teachers also stated the importance of having a neutral tone about eating according to various diets (e.g., vegetarian, or allergen-specific) to signal that different preferences are welcomed and to avoid drawing attention to it. In addition, some participants stated that they engage children in food-related activities to encourage healthy eating, and that children should be allowed to experiment with food, even if it becomes messy. Furthermore, teachers reported that some children are selective eaters and emphasized that they use terms such as ‘courageous taster’ or ‘being taste brave’ to boost confidence in children to try new vegetables and foods, as well as encouraging independent eating skills. However, participants observed that in some cultural contexts, children may be restrained from trying to eat by themselves from a young age. This was expressed as concerning, limiting the children’s motor skills in eating, chewing, and using cutlery. Another reported cultural difference was that some parents reasoned that sweets should be served celebrations at preschool.

## From policy to practice: supporting all teachers to promote healthy movement behaviours

There was consensus regarding the lack of policies or guidelines for physical activity in most preschools, and participants argued that a comprehensive, evidence-based strategy is needed to facilitate moderate-to-vigorous physical activity and to balance physical activity with sufficient rest. Participants emphasized the need for the preschool curriculum to offer clearer guidance on how to structure physical activity throughout the day, ensuring it becomes an integrated and natural part of the daily schedule, as this is currently lacking. They also noted that the curriculum prioritizes subjects that are preparatory for school over promotion of healthy lifestyle behaviours such as physical activity.

I think these questions [about healthy lifestyle behaviours] should form the foundation of the entire curriculum. […] We don’t need to mimic school with all the school subjects.—Principal 2.

One principal highlighted the value in using research reports as tools for creating an environment where physical activity is prioritized. Both principals and teachers underscored that support for physical activity should be included from a top-down approach, as directives from principals greatly influence the daily routines regarding children’s physical activity.

Both teachers and principals requested support to promote healthy lifestyle behaviours in children, especially among educators with low interest in physical activity. Suggestions included receiving encouragement and motivation through face-to-face support, inspirational lectures, education days, and training sessions followed by reoccurring reminders. Taken together, these factors were described as having the potential to increase awareness and motivation, and to empower teachers with a low interest in physical activity. Additionally, it was suggested that initiatives such as awareness weeks and sharing successful practices between preschools could boost teachers’ motivation for promoting physical activity.

I actually believe that it takes very little to create movement in children, and that it probably has more to do with the focus and knowledge of the staff. And maybe of course also… that the staff also understands that yes, this benefits the children. And not just the children. It benefits me as well.—Principal 5.

When discussing the content for a support tool, participants underscored the importance of offering practical, concrete advice and ready-made activity programs. Inclusion of specific activities such as dance, music, and aerobics as well as structured physical activity, preferably fitting into the existing preschool schedule, was suggested. Scheduling physical activity regularly, e.g., specific activities weekly, or awareness weeks quarterly, was highlighted as important. Some stated that scheduling was particularly essential for low-intensity physical activity as this does not occur spontaneously. In addition, easily accessible and evidence-based information on healthy lifestyle behaviours was highlighted as essential, and tips on field trips specific to the local area could further support the promotion of healthy movement behaviours.

There were conflicting views on using a digital support tool, e.g., an app or website. Some voiced that they would not use a digital tool due to time constraints and because they do not use screens in their teaching. Others believed that a digital tool could provide valuable evidence-based information, exercises and activities, making it easier to create physical activity opportunities both indoors and outdoors. It was stressed that such a support should be available to all preschools in Sweden to ensure equal opportunities for children.

People talk a lot about equal preschools, that it does not matter which area or which preschool you attend – you should receive the same thing. If there was an app that all preschools could access, then higher demands for equality could be reached. So that would be fantastic.—Teacher 6.

Finally, it was suggested that a digital tool could be extended to also target parents, as respondents addressed that parents do not receive sufficient guidance on healthy lifestyle behaviours from primary child healthcare, and that parents struggle to establish healthy routines at home.

## Discussion

The findings of this study highlight that preschool teachers perceived themselves and the preschool environment as important facilitators for promoting physical activity in the early years. Teacher involvement emerged as a key factor in motivating children to engage in physical activity, particularly in children who do not naturally enjoy it. Dialogue and collaboration between parents and preschool teachers was also important for fostering healthy lifestyle behaviour among children. Moreover, face-to-face support such as inspirational lectures or education days, which also could be complemented by a digital support tool, were seen as valuable for increasing awareness and motivation among educators to promote healthy lifestyle behaviours. Furthermore, such a combined approach was highlighted as particularly beneficial for supporting educators with lower engagement or interest in physical activity. Finally, participants perceived the sections on physical activity in the preschool curriculum as vague and difficult to interpret and expressed a need for a revision to better support the promotion of physical activity.

The results of this study are in line with a systematic review of qualitative literature by Hesketh et al., exploring barriers and facilitators for physical activity in children aged 0–6 years [23]. Similar to the current study, Hesketh et al. [23] found that childcare providers in preschool settings are essential for promoting physical activity in children. Not only the providers’ skills in engaging the children were highlighted as important, but also the providers’ own physical activity behaviours [23]. Interestingly, the role of teachers for preschool-aged children’s physical activity levels was also confirmed in a systematic review including objectively measured physical activity [24]. Tonge et al. found correlations between children’s physical activity and the presence and skills of educators, suggesting that active involvement and engagement may be important aspects, but found no studies investigating this [24].

Additionally, in a qualitative study by Ek et al. [25], Swedish preschool teachers underscored the impact of their own attitudes, interest and engagement in physical activity on children’s level and intensity of physical activity. Furthermore, the impact of the outdoor and indoor preschool environment on children’s physical activity has been addressed by previous research [23, 24, 26], emphasizing the importance of space as well as portable and fixed equipment for promoting physical activity, which is in line with the current study.

One perceived barrier for increasing children’s physical activity was the preschool curriculum and daily scheduling, which were described by the participants as more focused on school preparatory subjects rather than on physical activity. This aligns with Mak et al. [27] who reviewed strategies for promoting physical activity in the preschool setting and suggested that such prioritization may be explained by limited knowledge among decision makers about the importance of physical activity in young children. Indeed, the Swedish government recently called for the need to strengthen and clarify the guidance on physical activity in the preschool curriculum in order to emphasize the importance of integrating movement into daily routines [28, 29] and a new curriculum is planned to be published in 2025 [29]. Participants in the current study voiced that revising the sections on physical activity in the curriculum could be a way to motivate teachers to engage children in more physical activity, especially teachers with low interest in this area. Also, some respondents believed that moderate-to-vigorous physical activity is obtained when children play spontaneously, while others thought structured, teacher-led exercise is required. This highlights the importance of providing training and guidance for educators on what moderate-to-vigorous physical activity is and how it can be effectively integrated into everyday preschool routines. Furthermore, there was a lack of clear policies for physical activity among the participating preschools. This is noteworthy, as formal physical activity policies in Swedish preschools have been found to be associated with more active children, compared to preschools without policies [30]. Hence, a clear preschool curriculum as well as having local physical activity policies in place are essential to ensure an effective approach to promote healthy movement behaviours in young children. It is important to note that preschool systems across countries may differ and that the generalizability of the results regarding the Swedish curriculum and policies may be limited to countries with similar preschool systems as Sweden. However, having clear policies or guidelines for the preschool staff may contribute to healthier lifestyle behaviours in preschoolers, regardless of country, and we can also learn from each other’s systems.

With regards to screen time in the preschool setting, Ek et al. [25] found that teachers expressed concerns about the requirement to integrate screens into the daily routine, even before the publication of the WHO movement guidelines for young children [2]. Thus, although integrating screens into preschool routines was in accordance with the preschool curriculum at that time, teachers questioned the use of screens as they saw no educational benefit or believed it would be challenging for young children to learn how to use them [25]. Notably, this requirement has now been removed from the preschool curriculum, suggesting that these concerns were well-founded. Furthermore, participants in the present study emphasized that teaching children how to use screens is not the responsibility of preschools. It is also relevant to note that the planned update to the preschool curriculum will emphasize that preschools should be free from screen time [29].

In terms of sleep, participants highlighted that it is common for parents to stop allowing their child to nap at preschool, which was reported a barrier for supporting and promoting healthy sleep routines. This parental preference aligns with findings from a questionnaire-based study in Australian parents, which found that parents favoured putting their child to bed early over ensuring that their child naps at preschool [31]. Interestingly, teachers and principals in the current study believed that downtime is crucial for children’s recovery and described it as a natural part of the preschool schedule. Similarly, Hesketh et al. [23] found that childcare providers repeatedly stressed the importance of children having downtime. While other sedentary activities during downtime, such as reading, are known as being important for children’s development, both studies highlighted the significance of downtime itself. Currently, the WHO guidelines [2] include napping as part of the 10–13 h of daily sleep recommended; however, the benefits of downtime in the preschool setting are not clearly supported by current evidence. Therefore, more research is needed on possible advantages of downtime for recovery in the preschool setting.

Although it was emphasized that selective eating occurs to a small extent and that it is important to encourage all children to try new vegetables and foods, eating behaviours in the preschool setting were not considered challenging. Similar to our findings, qualitative data from early childhood teachers in Portugal, where preschool meals are also provided, highlighted that teachers are important role models for healthy eating behaviours in children [32]. They also expressed that the healthy meals provided at preschools may compensate for poor eating habits at home [32]. Together, this suggests that when healthy meals are provided within the preschool setting, teachers are not concerned about children’s eating behaviours in the preschool environment.

Participants in the current study expressed interest in being supported in promoting healthy movement and eating behaviours through inspirational lectures. Also, having a library of activities easily accessible, for instance in an app, was desired, especially to facilitate physical activity promotion for teachers with a low interest in physical activity. In the study by Ek et al. [25], teachers were also interested in an app to promote physical activity; however, they emphasized the particular need for it in situations where spontaneous activity is restricted, such as on days spent mostly indoors or when space is limited. Taken together, this highlights the need for a support that offers concrete, practical guidance tailored to the preschool setting.

### Strengths and limitations

This study has several strengths. First, performing the interviews using video calls enabled participation from multiple regions in Sweden, increasing the geographic range of the sample. Additionally, conducting the interviews in a familiar setting may have encouraged participants to speak more openly about their experiences and perspectives [33]. The study was further strengthened by the inclusion of participants from a wide range of preschool contexts across Sweden, capturing experiences from different settings. Furthermore, participants’ levels of education and years of work experience varied, contributing to a broad range of perspectives. A potential limitation is related to sample characteristics, as it is possible that the teachers and principals agreeing to participate had a specific interest in healthy lifestyle behaviours. Hence, perspectives from teachers and principals that are less engaged in this topic may be underrepresented. However, as most of those invited chose to participate (15 out of 18), the risk of selection bias appears small. Nevertheless, this study provides valuable insights and perspectives of preschool teachers and principals on promoting healthy lifestyle behaviours, and findings contribute to the evidence on how to best support preschools in fostering healthy lifestyle behaviours.

To ensure the trustworthiness of this study, established criteria for qualitative research were followed, including credibility, dependability, and transferability [21]. Credibility was strengthened through data triangulation using purposive sampling, ensuring that participants represented diverse perspectives (e.g. various regions, urban and rural locations, and women and men). Additionally, a wide age range (31–64 years), and participant triangulation via including two levels of respondents (principals and teachers) provided nuanced experiences. The semi-structured interview guide ensured consistency across interviews while also leaving room for individualized questions. Additionally, credibility was strengthened by investigator triangulation, as researchers with different expertise (nutrition and physiotherapy) developed the interview guide and analysed the data. Dependability was accomplished by having the same researcher conduct all interviews, ensuring consistency. The interviews were audio-recorded to assure no details were lost. Transferability of the findings was supported through a detailed study description of participants and the preschool settings. Finally, to further increase trustworthiness, COREQ [20] was used to confirm that all necessary aspects of the study were reported.

### Implications

The findings of this study indicate the need for a clearer curriculum and preschool policies to better support physical activity in the preschool setting. Notably, the Swedish government has announced that an updated preschool curriculum will be published in 2025, with a strengthened emphasis on increasing physical activity and reducing screen time. Our results support this direction, as participants expressed a need for more concrete and practical guidance to promote healthy movement behaviours. The results also indicate a need for evidence-based support tailored to the preschool setting as the level of encouragement is highly dependent on individual knowledge and interests among preschool teachers. Decision makers should include clear guidance on supporting healthy movement behaviours in the preschool curriculum and local policies to ensure consistency across preschools. In addition, we suggest that the curriculum highlight the importance of physical activity in early childhood for long-term health. Strengthening this may help boost teachers’ motivation and the prioritization of physical activity in the preschool setting.

Strategies for effectively integrating physical activity into daily routines are also warranted. Workshops, lectures, and providing teachers with concrete approaches to encourage physical activity should be prioritized in such strategies. Furthermore, a digital tool for promoting healthy lifestyle behaviours, combined with face-to-face support, align with needs and preferences of preschool teachers and principals, proposing a broad support to fit all preschool settings. Preferably, such a tool should be developed through a co-design process [34] together with teachers, principals and parents. In addition, encouraging collaboration between preschools and parents is essential in shaping healthy lifestyle behaviours and future initiatives should aim to strengthen the communication between them to ensure a unified approach towards healthy lifestyle behaviours. Taken together, addressing these gaps is crucial to ensure that all children, receive the best possible opportunities for healthy movement behaviours throughout childhood.

## Conclusion

The findings propose that a digital tool in combination with face-to-face support may be an effective strategy for promoting physical activity and other healthy lifestyle behaviours in the preschool setting. However, to ensure that healthy lifestyle behaviours are prioritized and integrated into preschools’ daily routines, support is also needed on the policy level. This includes clear and concrete guidance within the preschool curriculum and policy documents, providing educators with the tools and resources needed to confidently promote healthy lifestyle behaviours in preschools.

## Supplementary Information

Below is the link to the electronic supplementary material.

Additional file 1: Interview guide for preschool teachers and principals on their perceptions of children’s healthy lifestyle behaviours and their preferences for a potential digital support to promote these behaviours in preschool aged children

## Data Availability

The material in this study is not publicly accessible but can be obtained from the corresponding author upon reasonable request.
