# Supplementary material for: Promoting healthy lifestyle behaviours in the preschool setting: perceptions and needs of teachers and principals
Source: BMC Public Health. 2025 Sep 9;25:3042. doi: 10.1186/s12889-025-24379-4 (PMC12418632; doi:10.1186/s12889-025-24379-4)
Supplement: Supplementary file 1 — Additional file 1: Interview guide for preschool teachers and principals on their perceptions of children’s healthy lifestyle behaviours and their preferences for a potential digital support to promote these behaviours in preschool aged children [file 12889_2025_24379_MOESM1_ESM.pdf]

Interview guide

Questions to preschool principals and teachers about children's movement and eating behaviour in the preschool setting and a digital tool aiming at promoting healthy lifestyle behaviour

How would you describe physical activity (PA) in children 3-5 years old?

When and where would you say that children in this age are physically active?

What *type* of PA do you think children of this age need and why?

How *much* PA do you think they need?

Why – can you describe/explain more?

Does the preschool have any policies or guidelines for children's PA, screen time, sleep and eating behaviours? What do they look like?

Are the guidelines available for you as educators and for parents and the public?

What would make it easier to reach the guidelines?

What are the main obstacles?

What types of activities that include physical activity do you have at the preschool?

How do you perceive these activities?

Is there anything else you can do/offer?

What types of activities do you think they need more of?

What do you think facilitates PA in preschool children?

What barriers to being physically active do you think there are?

How could children be more physically active at preschool than they currently are?

What can *you* do to increase PA at your preschool?

When is it difficult to make time for or incorporate PA at preschool?

How do you handle it?

In what ways do you think the preschool environment contributes to children's PA needs?

How does *your* preschool encourage PA (difference between seasons)?

What would need to be different for you or your preschool to encourage PA more often?

If you could change one thing in your preschool to promote more PA, what would it be?

What are your thoughts about PA in higher intensities (i.e., PA that generates higher pulse)?

Are there any differences from previous years?

What do you think would facilitate higher intensity PA?

What type of resources/support would that require?

Can you elaborate/explain more?

Interview guide

In preschoolers, what are your thoughts about:

Screen time? Sleep? Healthy eating behaviours?

What do you think is most important for a preschool child when it comes to food?

What can be done to improve children's eating behaviours in preschool?

How do you perceive preschool staff's knowledge of healthy eating behaviours for children?

What are the most common problems regarding food in preschool? (What are your thoughts on the food offered at preschool?)

What are your thoughts about using an app to promote healthy movement and eating behaviours in preschoolers?

How could such an app be used within the preschool setting?
What would facilitate the use of such an app?
What barriers can you see with introducing/using such an app?
When would *you* use such an app?

If you were to create such an app, what would it look like?

What features/content would you want such an app to have?
If the support were not delivered via an app, how would you prefer it to be delivered?

Do you have anything you would like to add?
